# Performance of Two Commercial Assays for the Detection of Serum *Aspergillus* Galactomannan in Non-Neutropenic Patients

**DOI:** 10.3390/jof8070741

**Published:** 2022-07-18

**Authors:** Rodrigo Almeida-Paes, Marcos de Abreu Almeida, Priscila Marques de Macedo, Diego H. Caceres, Rosely Maria Zancopé-Oliveira

**Affiliations:** 1 Laboratório de Micologia, Instituto Nacional de Infectologia Evandro Chagas, Fundação Oswaldo Cruz, Rio de Janeiro 21040-900, RJ, Brazil; rodrigo.paes@ini.fiocruz.br (R.A.-P.); marcos.almeida@ini.fiocruz.br (M.d.A.A.); 2 Laboratório de Pesquisa Clínica em Dermatologia Infecciosa, Instituto Nacional de Infectologia Evandro Chagas, Fundação Oswaldo Cruz, Rio de Janeiro 21040-900, RJ, Brazil; priscila.marques@ini.fiocruz.br; 3 Immuno-Mycologics (IMMY), Norman, OK 73069, USA; diego-caceres@immy.com or; 4 Center of Expertise in Mycology Radboudumc/CWZ, 6525GA Nijmegen, The Netherlands; 5 Studies in Translational Microbiology and Emerging Diseases (MICROS) Research Group, School of Medicine and Health Sciences, Universidad del Rosario, Bogota 1653, Colombia

**Keywords:** *Aspergillus* antigen, diagnosis, ELISA, lateral flow

## Abstract

Besides the relevance of aspergillosis in neutropenic patients, this mycosis has gained significance among non-neutropenic patients in last years. The detection of *Aspergillus* galactomannan has been used for aspergillosis diagnosis and follow-up in neutropenic patients. This study evaluated the applicability of two commercial tests for galactomannan detection in non-neutropenic patients with different clinical forms of aspergillosis. Serum samples from patients with chronic pulmonary aspergillosis, aspergilloma, invasive aspergillosis, and COVID-19 associated pulmonary aspergillosis were evaluated using the IMMY sōna AGM lateral flow assay and the Bio-Rad Platelia sandwich ELISA. Serum specimens from patients with tuberculosis, histoplasmosis, paracoccidioidomycosis, and from healthy individuals were used as controls. The Bio-Rad Platelia sandwich ELISA presented greater sensitivity, whereas the IMMY sōna AGM lateral flow assay presented greater specificity. The accuracies of the tests were similar, as demonstrated by a receiver operator characteristic analysis. Moreover, the best cut-off values determined by this analysis were closer to that recommended by both manufacturers for neutropenic patients. The galactomannan indexes determined by different methodologies were strongly related, and a substantial agreement was observed between results. Both tests can be used in non-neutropenic patients with the cut-off values defined by the manufacturers. *Histoplasma* cross-reactions may occur in areas where histoplasmosis is endemic.

## 1. Introduction

The genus *Aspergillus* is a polyphyletic group of airborne fungi that is also found in soil samples and decaying organic matter. The major propagation form of these fungi is through the dispersion of conidia, asexual reproduction structures, that are easily dispersed through the air [1]. Animals inhale these conidia constantly, but their innate immune response impairs fungal invasion [2]. However, if the fungus finds a susceptible host, a wide spectrum of diseases can occur [1].

Therefore, aspergillosis is an opportunistic mycosis that usually affects patients with immunodeficiencies, e.g., prolonged neutropenia, organ transplantation, and those under steroid or immunosuppressive treatments [3]. Depending on some underlying status of the patients, they can develop hypersensitivity reactions, such as severe asthma with fungal sensitization and allergic bronchopulmonary aspergillosis, structural diseases such as aspergilloma and chronic pulmonary aspergillosis, or invasive manifestations, such as invasive pulmonary aspergillosis and invasive bronchial aspergillosis [1].

The coronavirus disease 2019 (COVID-19) has brought a new group of critically ill patients at risk for severe *Aspergillus* infection. In fact, among a series of secondary infections that can affect these patients, coronavirus-associated pulmonary aspergillosis (CAPA) emerges as an important cause of morbidity and mortality [4]. A CAPA overall incidence of 13.5% has been described, starting from day 4 to 15 after ICU admission, and a mortality rate as high as 48.4% [5]. Nevertheless, the real magnitude of this problem is unknown due to the limitations of aspergillosis diagnosis.

Aspergillosis diagnosis is challenging because the isolation of an *Aspergillus* colony in culture from clinical specimens does not necessarily indicate infection. First, the host may present colonization, not infection, of the upper airways. In addition, contamination of the biological specimen during clinical collection or laboratory processing by these airborne fungi may occur, which makes an association of clinical, radiological, and microbiological features necessary for an accurate and reliable diagnosis [6]. One of the most used aspergillosis diagnostic assays is the detection of galactomannan in serum or bronchoalveolar lavage (BAL) from patients. This test was extensively validated for neutropenic patients under treatment for hematological malignancies or in hematopoietic stem cell transplantation recipients without antifungal prophylaxis, presenting elevated sensitivity (range: 67–100%) and specificity (range: 86–99%) [1,7]. However, there is a paucity of studies about the performance of galactomannan detection in non-neutropenic patients, especially those with unknown pulmonary infections. Since several fungi can produce galactomannan, cross-reactions can occur, such as those reported with *Histoplasma capsulatum*, *Paracoccidioides brasiliensis*, and *Cryptococcus neoformans*. The mechanisms of these false-positive results are unknown [8].

For decades, *Aspergillus* galactomannan (AGM) testing was performed in a sandwich enzyme-linked immunosorbent assay (ELISA) format using a monoclonal antibody of high affinity and avidity for lateral galactofuranose residues of the whole biomarker [9]. Most recently, a lateral flow assay (LFA) was developed, which uses two monoclonal antibodies against different epitopes of *Aspergillus* galactomannan [10].

Due to the increased galactomannan testing in non-neutropenic patients, especially after the COVID-19 pandemic emergence, and the paucity of studies in this population, we evaluated the performance of the sōna AGM lateral flow assay (LFA) (IMMY, Norman, OK, USA) for the diagnosis of various clinical presentations of aspergillosis. In addition, we determined the specificity of this test using serum samples in some diseases not evaluated previously and performed a comparison with the Bio-Rad Platelia sandwich ELISA (Bio-Rad Laboratories, Hercules, CA, USA).

## 2. Materials and Methods

### 2.1. Study Design, Patients, and Sera

**Figure 1:**
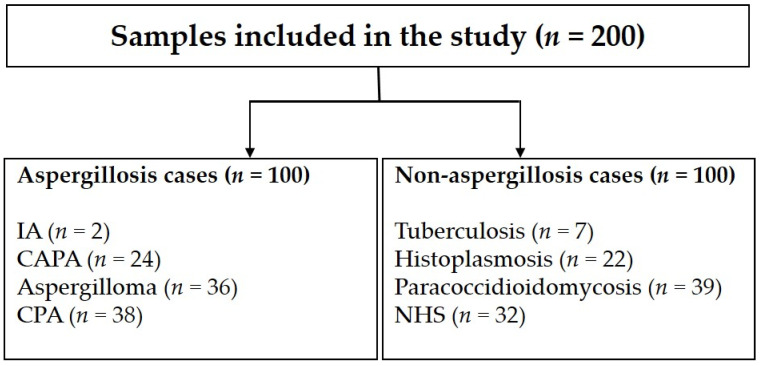
Flowchart representing the serum samples included in this study. In total, 200 samples were studied: 100 from patients with different aspergillosis clinical forms and 100 from individuals with other infectious diseases and healthy individuals. IA = invasive aspergillosis; CAPA = COVID-19-associated pulmonary aspergillosis; CPA = chronic pulmonary aspergillosis; NHS = sera from healthy individuals.

We conducted a retrospective, case control study involving 168 serum samples collected for routine diagnosis of pulmonary infections at the Evandro Chagas National Institute of Infectious Diseases (INI), Fiocruz, Rio de Janeiro, Brazil. The homologous group included patients with proven or probable aspergillosis. Patients with different clinical forms of the disease were included, which consist of chronic pulmonary aspergillosis (*n* = 38), aspergilloma (*n* = 36), invasive aspergillosis (*n* = 2), and CAPA (*n* = 24). The heterologous group included patients with tuberculosis (*n* = 7), histoplasmosis (*n* = 22), and paracoccidioidomycosis (*n* = 39). Additionally, 32 serum samples collected from healthy blood donors from the same region were included as controls. The 200 serum samples included in this study (Figure 1) were stored at −80 °C in our laboratory up to use. Two professionals evaluated the samples after their blinding. The Institutional Research Ethics Committee of INI, Fiocruz approved the galactomannan testing in all samples (CAAE 31578820.9.0000.5262).

### 2.2. Case Definitions

We used the definitions of the European Organization for Research and Treatment of Cancer and the Mycoses Study Group Education and Research Consortium (EORTC/MSGERC) to define aspergillosis certainty in each patient [11]. Aspergillosis clinical forms were classified according to the spectrum of *Aspergillus* diseases reviewed by Latge and Chamilos [1]. CAPA was defined following ISHAM guidelines for case definition [12]. Proven tuberculosis and histoplasmosis were defined after culture isolation of *Mycobacterium tuberculosis* and *H. capsulatum*, respectively, from any clinical sample from the patients. Proven paracoccidioidomycosis was defined after the culture isolation of *P. brasiliensis* or its observation in histopathologic or direct examination tests. The individuals with tuberculosis and healthy individuals did not present evidence of fungal infections.

### 2.3. IMMY Sōna AGM Lateral Flow Assay

We followed the manufacturer’s instructions to perform the LFA in all serum samples. The cube reader compatible with sōna AGM LFA (IMMY, Norman, OK, USA) was used to determine the galactomannan index (GMI) of each sample. The GMI of 0.5 suggested by the manufacturer was the initial cut-off to classify samples as negative (GMI < 0.5) or positive (GMI ≥ 0.5).

### 2.4. Bio-Rad Platelia Sandwich ELISA

Again, we strictly followed the manufacturer’s instructions to detect galactomannan in the samples. Optical densities were determined using a SpectraMax Plus reader (Molecular Devices, San Jose, CA, USA). A GMI of 0.5 suggested by the manufacturer was the initial cut-off to classify samples as negative (GMI < 0.5) or positive (GMI ≥ 0.5).

### 2.5. Statistical Analyses

Clinical performance of both assays in the studied population was verified using 2 × 2 tables to calculate sensitivity, specificity, accuracy, positive and negative likelihood ratios, and diagnostic odds ratio of the tests. Due to the diversity of individuals in the control group, including individuals with other fungal infections with a high risk of cross reactivity, we evaluated the specificity of these assay overall, and by splitting the control group into three subgroups. A receiver operating characteristic (ROC) analysis was used to determine the overall performance of the tests in the studied population, and to identify optimal positivity thresholds to enhance specificity, sensitivity, and accuracy of the tests [13]. To check the qualitative agreement between the two commercial kits studied, we applied the Cohen’s kappa coefficient, whereas the Spearman’s rank correlation coefficient was used to determine the quantitative agreement. Median values were compared using the Mann–Whitney test for pairwise analysis or the Kruskal–Wallis when comparing multiple median values of different groups. GraphPad Prism 8.4 was used for statistics and a *p* < 0.05 was considered significant.

## 3. Results

### 3.1. Overall Performance of Individual Assays

Table 1 depicts the performance of both tests in the serum samples studied using the cut-off values suggested by the manufacturer of each test. The IMMY sōna AGM lateral flow assay presented higher specificity and the Bio-Rad Platelia *Aspergillus* sandwich ELISA higher sensitivity. The Wilcoxon signed rank test revealed a strong relationship between GMI values obtained by both methodologies (*p* < 0.0001), and a significant effective pairing, as revealed by the Spearman test (*r_s_* = 0.7209, *p* < 0.0001).

### 3.2. Performance of the Assays in Individuals with Aspergillosis

#### 3.2.1. IMMY Sōna AGM Lateral Flow Assay

**Figure 2:**
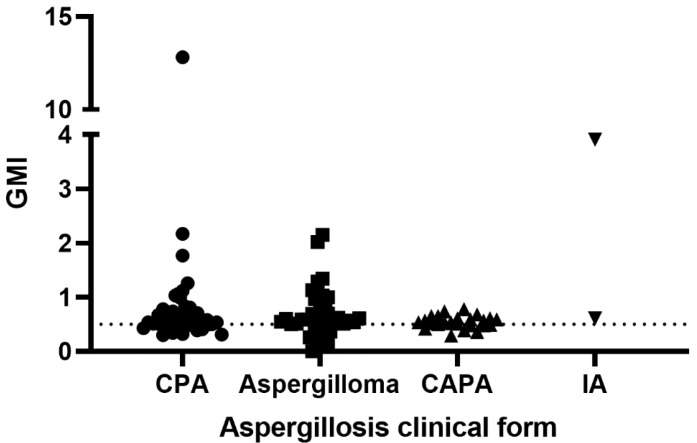
Galactomannan indexes of 100 patients with aspergillosis tested with the IMMY sōna AGM lateral flow assay. The dotted line represents the cut-off recommended by the manufacturer. GMI = galactomannan index; CPA = chronic pulmonary aspergillosis; CAPA = COVID-19-associated pulmonary aspergillosis; IA = invasive aspergillosis.

Figure 2 presents the GMI values obtained for each group of aspergillosis patients when tested with the IMMY sōna AGM lateral flow assay. In brief, 26% of samples presented false-negative results. They include samples from 10 patients with chronic pulmonary aspergillosis, nine patients with aspergilloma, and seven with CAPA. According to the Kruskal–Wallis test, there is no significant difference between these groups (*p* = 0.2503).

#### 3.2.2. Bio-Rad Platelia Sandwich ELISA

**Figure 3:**
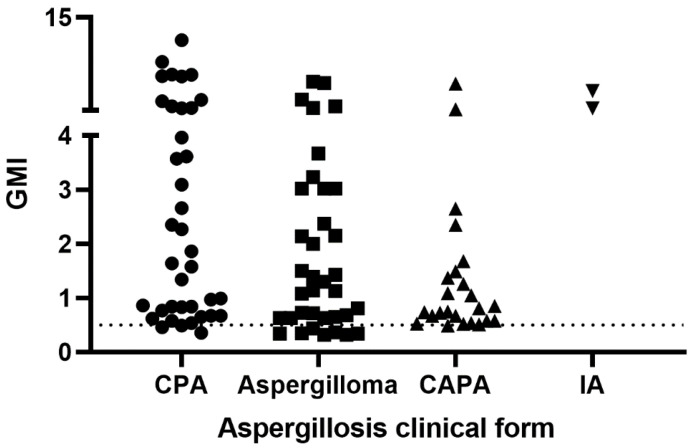
Galactomannan indexes of 100 patients with aspergillosis tested with the Bio-Rad Platelia sandwich ELISA. The dotted line represents the cut-off recommended by the manufacturer. GMI = galactomannan index; CPA = chronic pulmonary aspergillosis; CAPA = COVID-19-associated pulmonary aspergillosis; IA = invasive aspergillosis.

Figure 3 shows the GMI observed for each group of aspergillosis patients when tested with the Bio-Rad Platelia sandwich ELISA. Eleven patients presented false-negative results: three with chronic pulmonary aspergillosis, seven with aspergilloma, and one with CAPA. A significant difference was observed among the GMI of the groups of patients with aspergillosis (*p* = 0.0266).

### 3.3. Performance of the Assays in Individuals without Aspergillosis

#### 3.3.1. IMMY Sōna AGM Lateral Flow Assay

**Figure 4:**
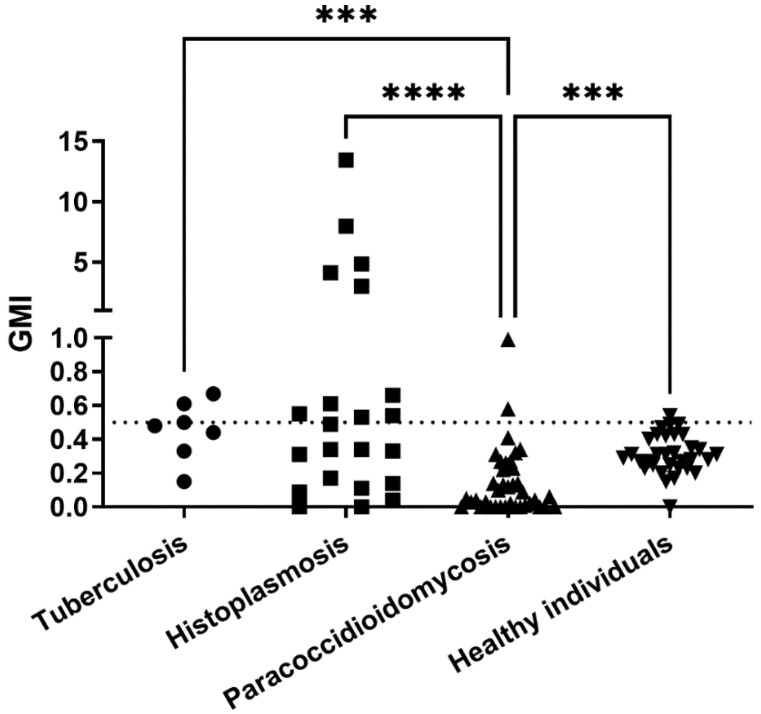
Galactomannan indexes of 100 individuals without aspergillosis tested with the IMMY sōna AGM lateral flow assay. The dotted line represents the cut-off recommended by the manufacturer. Significant differences, tested by the Dunn’s multiple comparison test, are represented. *** *p* < 0.001, **** *p* <0.0001, GMI = galactomannan index.

Figure 4 presents the GMI values obtained for each group of patients without aspergillosis when tested with the IMMY sōna AGM lateral flow assay. In total, 16 of 100 serum samples presented false-positive results. They include samples from 3 patients with tuberculosis, 2 with paracoccidioidomycosis, 10 patients with histoplasmosis, and 1 healthy blood donor. Therefore, specificities for each subgroup were as follows: 95% for paracoccidioidomycosis, 55% for histoplasmosis, and 90% for the “true” control group. Patients with histoplasmosis yielded the highest GMI values, five of them with GMI > 1.0, while all patients from the other three groups yielded GMI < 1.0. The Kruskal–Wallis test showed a significant difference between GMI means of the groups (*p*< 0.0001).

#### 3.3.2. Bio-Rad Platelia Sandwich ELISA

**Figure 5:**
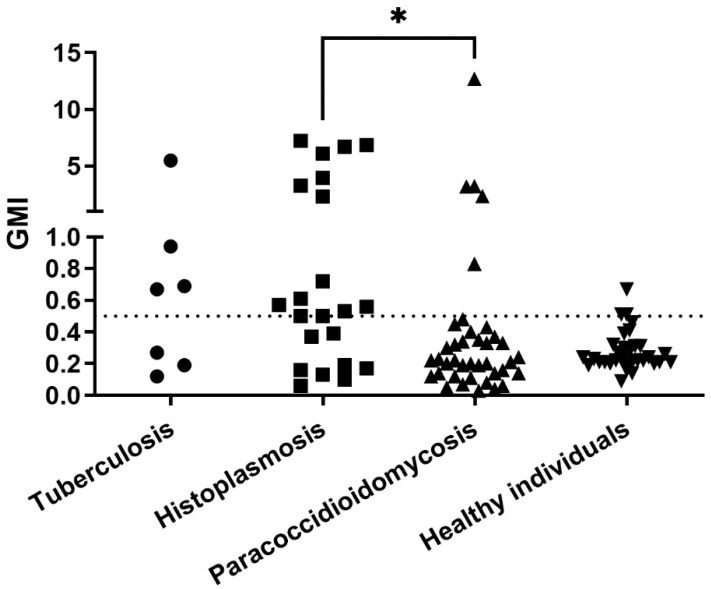
Galactomannan indexes of 100 individuals without aspergillosis tested with the Bio-Rad Platelia sandwich ELISA. The dotted line represents the cut-off recommended by the manufacturer. Significant difference, tested by the Dunn’s multiple comparison test, is represented. * *p* < 0.05, GMI = galactomannan index.

Figure 5 presents the GMI values of each group of patients without aspergillosis when tested with the Bio-Rad Platelia sandwich ELISA. In total, 25 of 100 serum samples presented false-positive results. They include samples from 4 patients with tuberculosis, 5 with paracoccidioidomycosis, 13 with histoplasmosis, and 3 healthy blood donors. The specificities for the paracoccidioidomycosis, histoplasmosis, and “true” control group were 87%, 41%, and 82%, respectively. The Kruskal–Wallis test showed a significant difference between GMI of the groups (*p* = 0.0113).

### 3.4. Receiver Operator Characteristic (ROC)-Based Cut-Off Calculations

**Figure 6:**
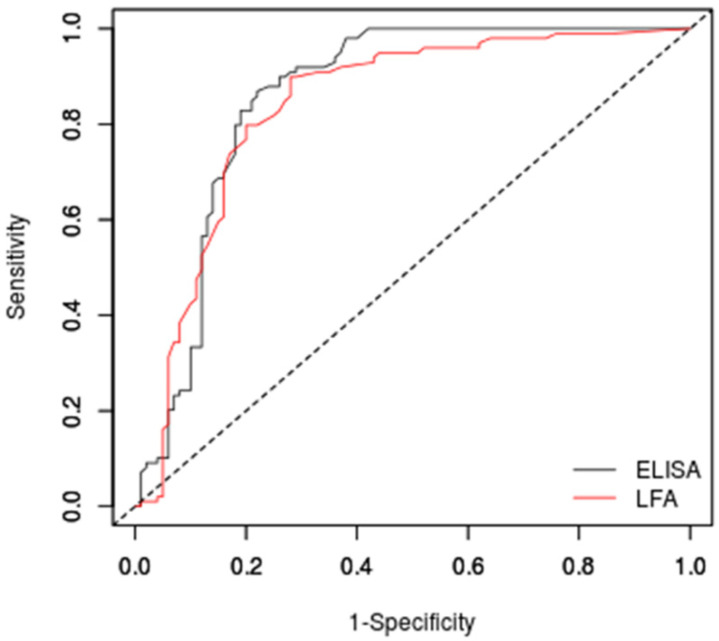
Receiver operator characteristic curves of the two commercial tests evaluated in this study. ELISA = Bio-Rad Platelia sandwich ELISA; LFA = IMMY sōna AGM lateral flow assay.

Figure 6 presents the ROC curves of both tests in the studied population. The area under the ROC curve was 0.838 (95% CI: 0.779–0.897) for the IMMY sōna *Aspergillus* GM lateral flow assay and 0.858 (95% CI: 0.802–0.915) for the Bio-Rad Platelia *Aspergillus* sandwich ELISA.

According to the ROC analysis, the best cut-off value for the IMMY sōna *Aspergillus* GM lateral flow assay to maximize accuracy was 0.36, to minimize the most frequent error was 0.48, and for similar sensitivity/specificity values was also 0.48. Table 2 presents the performance of this test using these cut-off values.

For the Bio-Rad Platelia *Aspergillus* sandwich ELISA, the best cut-off to maximize accuracy was 0.52, to minimize the most frequent error was 0.58, and for similar sensitivity/specificity values was 0.61. Table 3 presents the performance of this test using these cut-off values.

### 3.5. Comparison between Assays

The Bonferroni method was used to compare the area under the two ROC curves presented in Figure 6. This test showed no statistical difference between them (*p* = 0.5669). A categorical agreement analysis using the kappa test showed a substantial agreement between the two methods when the manufacturer cut-off was used, with a kappa value of 0.675 (95% CI: 0.577–0.773). The agreements of the tests for each subgroup herein studied were as follows: 76% for chronic pulmonary aspergillosis patients, 83% for aspergilloma patients, 67% for CAPA patients, 100% for invasive aspergillosis patients, 92% for paracoccidioidomycosis patients, 86% for histoplasmosis patients, and 92% for the “true” control group.

## 4. Discussion

Invasive aspergillosis is, indeed, a major health problem, especially in patients with acute leukemia and recipients of hematopoietic stem cell transplantation [14]. Detection of circulating galactomannan is a test of choice for invasive aspergillosis in these patients, with both ELISA and LFA commercial kits validated in clinical studies [10,15]. Despite the increasing number of non-neutropenic patients at-risk for *Aspergillus* infection in recent years [1], clinical validation and comparative studies in this population with the IMMY sōna AGM LFA are missing [16].

This new commercial assay was previously evaluated for diagnosis of aspergillosis using serum samples from neutropenic patients [10]. Sensitivity and specificity for this group of patients were higher than that observed in this study. The higher sensitivity in neutropenic patients may be associated to an impaired immune response against the fungus, yielding higher amounts of circulating antigen. In patients with CPA and aspergilloma, the lack of an invasive presentation and the limited hyphal growth could affect galactomannan circulation, leading to a serum concentration below the limit of detection of the AGM LFA. The higher specificity for the previous study may be associated with the lack of patients with other mycotic pulmonary infections in the control group. The best cut-off value found in the study with neutropenic patients was validated as 0.5, the value suggested by the manufacturer, and supported by the ROC analysis that showed close cut-off values for similar sensitivity/specificity values and to minimize the most frequent error. In this study, to achieve the highest accuracy, that is minimizing erroneous results, the cut-off is suggested to decrease to 0.36, allowing the detection of lower galactomannan levels. Equally, for the Platelia *Aspergillus* sandwich ELISA, the highest accuracy cut-off observed in this study is close to that recommended by the manufacturer to be used in neutropenic patients. Previous studies revealed that the sensitivity of the ELISA in non-neutropenic patients with invasive pulmonary aspergillosis using this cut-off was very low [17,18] An explanation for this difference may be the low number of patients with invasive aspergillosis in our study. Therefore, we suggest that the 0.5 cut-off value suggested by the manufacturer of both tests can also be used on non-neutropenic patients.

Some studies show that the well-established Platelia *Aspergillus* sandwich ELISA can present cross-reactions with samples from patients with cryptococcosis [19], invasive *Fusarium* infection [20], blastomycosis [21], and particularly paracoccidioidomycosis and histoplasmosis [8]. For this latter disease, this method is even suggested for diagnosis in some cases [22]. This study showed that the IMMY sōna AGM LFA presented a slightly higher specificity, which means less cross-reactions than the sandwich ELISA. This was particularly true for paracoccidioidomycosis, where most cross-reactions observed using the Platelia kit were absent when the IMMY sōna AGM LFA was used. Patients with histoplasmosis yielded the highest GMI values on both tests, corroborating the antigenic similarity between galactomannans of *H. capsulatum* and *Aspergillus* spp. previously described [22,23,24,25]. The three patients with tuberculosis with positive results in the LFA did not have concomitant diagnosis of aspergillosis at the time of sample collection. In addition, they were also positive in the sandwich ELISA. Since pulmonary tuberculosis and *Aspergillus* coinfection is relatively common [26] and both diseases share radiologic findings [27,28], we cannot rule out the possibility of this coinfection in the three patients with proven tuberculosis and positive LFA results.

Some limitations of this study include the lack of some clinical data due to its retrospective nature. In addition, this study was carried out in a referral center that receives samples from different hospitals, which makes the retrieval of patient data difficult. The unknown prevalence of aspergillosis in non-neutropenic patients [1] prevented a precise sample size calculation and determination of positive and negative predictive values. Finally, the unavailability of bronchoalveolar lavage samples of the patients restricted a comparison between the accuracy of the test with the two specimen types that can be tested with these commercial assays. In fact, BAL is superior to serum in the detection of invasive aspergillosis in non-neutropenic patients by the ELISA method [18].

This study provides evidence that, even with lower sensitivity and specificity values than those observed when serum samples from neutropenic patients are tested [10], the IMMY sōna AGM LFA commercial kit is a useful tool for aspergillosis diagnosis in non-neutropenic patients, with a similar performance than that observed for the sandwich ELISA format used for decades in invasive aspergillosis diagnosis. Cross-reactions with other infectious diseases can occur, especially with histoplasmosis and clinicians must pay attention to positive results in non-neutropenic patients from areas of histoplasmosis endemicity.

## Figures and Tables

**Table 1:** Clinical performance of the IMMY sōna *Aspergillus* GM lateral flow assay and the Bio-Rad Platelia sandwich ELISA when testing serum samples from non-neutropenic patients with pulmonary infections and control healthy individuals based on the cut-off values determined by the manufacturer of each test.

Performance Parameter	Lateral Flow Assay		Sandwich ELISA	
	Value	95% CI ^a^	Value	95% CI
Sensitivity	74%	64.27–82.26%	89%	81.17–94.38%
Specificity	84%	75.32–90.57%	75%	65.34–83.12%
Accuracy	79%	72.69–84.43%	82%	75.96–87.06%
Positive Likelihood Ratio	4.62	2.91–7.35	3.56	2.52–5.03
Negative Likelihood Ratio	0.31	0.22–0.44	0.15	0.08–0.26

^a^ CI = confidence interval.

**Table 2:** Clinical performance of the IMMY sōna *Aspergillus* GM lateral flow assay when testing serum samples from non-neutropenic patients with pulmonary infections and control healthy individuals using ROC based cut-off values.

Performance Parameter	ROC Based Cut-Off Value		
	0.36	0.48	0.48
Sensitivity (95% CI)	90% (82.2–95%)	80% (70.8–87.3%)	80% (70.8–87.3%)
Specificity (95% CI)	72% (62.1–80.5%)	80% (70.8–87.3%)	80% (70.8–87.3%)
Accuracy (95% CI)	81% (74.9–86.2%)	80% (73.8–85.3%)	80% (73.8–85.3%)
Positive Likelihood Ratio (95% CI)	3.211 (2.329–4.427)	3.990 (2.663–5.978)	3.990 (2.663–5.978)
Negative Likelihood Ratio (95% CI)	0.140 (0.077–0.256)	0.253 (0.169–0.378)	0.253 (0.169–0.378)

**Table 3:** Clinical performance of the Bio-Rad Platelia sandwich ELISA when testing serum samples from non-neutropenic patients with pulmonary infections and control healthy individuals using ROC based cut-off values.

Performance Parameter	ROC Based Cut-Off Value		
	0.52	0.58	0.61
Sensitivity (95% CI)	87% (78.8–92.9%)	83% (74.2–89.8%)	80% (70.8–87.3%)
Specificity (95% CI)	78% (68.6–85.7%)	81% (71.9–88.2%)	81% (71.9–88.2%)
Accuracy (95% CI)	82.5% (76.5–87.5%)	82% (76–87.1%)	80,5% (74.3–85.8%)
Positive Likelihood Ratio (95% CI)	3.955 (2.713–5.764)	4.368 (2.887–6.611)	4.211 (2.777–6.385)
Negative Likelihood Ratio (95% CI)	0.167 (0.099–0.280)	0.210 (0.135–0.327)	0.247 (0.165–0.370)

## Data Availability

The data presented in this study are available within the article.
